# Effects of Intermittent Administration of Parathyroid Hormone (1-34) on Bone Differentiation in Stromal Precursor Antigen-1 Positive Human Periodontal Ligament Stem Cells

**DOI:** 10.1155/2016/4027542

**Published:** 2016-03-16

**Authors:** Xiaoxiao Wang, Yanlan Wang, Xubin Dai, Tianyu Chen, Fanqiao Yang, Shuangye Dai, Qianmin Ou, Yan Wang, Xuefeng Lin

**Affiliations:** ^1^Guanghua School of Stomatology, Sun Yat-sen University, Guangdong Provincial Key Laboratory of Stomatology, Guangzhou 510080, China; ^2^School and Hospital of Stomatology, Wenzhou Medical University, Wenzhou 325035, China

## Abstract

Periodontitis is the most common cause of tooth loss and bone destruction in adults worldwide. Human periodontal ligament stem cells (hPDLSCs) may represent promising new therapeutic biomaterials for tissue engineering applications. Stromal precursor antigen-1 (STRO-1) has been shown to have roles in adherence, proliferation, and multipotency. Parathyroid hormone (PTH) has been shown to enhance proliferation in osteoblasts. Therefore, in this study, we aimed to compare the functions of STRO-1(+) and STRO-1(−) hPDLSCs and to investigate the effects of PTH on the osteogenic capacity of STRO-1(+) hPDLSCs in order to evaluate their potential applications in the treatment of periodontitis. Our data showed that STRO-1(+) hPDLSCs expressed higher levels of the PTH-1 receptor (PTH1R) than STRO-1(−) hPDLSCs. In addition, intermittent PTH treatment enhanced the expression of PTH1R and osteogenesis-related genes in STRO-1(+) hPDLSCs. PTH-treated cells also exhibited increased alkaline phosphatase activity and mineralization ability. Therefore, STRO-1(+) hPDLSCs represented a more promising cell resource for biomaterials and tissue engineering applications. Intermittent PTH treatment improved the capacity for STRO-1(+) hPDLSCs to repair damaged tissue and ameliorate the symptoms of periodontitis.

## 1. Introduction

Periodontitis is the most common cause of irreversible destruction of periodontal tissue and tooth loss in adults worldwide. However, conventional periodontal therapy for the treatment of periodontal tissue damage does not induce the regeneration of periodontal supporting tissue [1, 2]. Therefore, the development of regenerative therapies for the treatment of periodontal disease has become a major challenge [3].

Several studies have suggested that mesenchymal stem cells (MSCs) may represent promising therapies for the functional repair of defect and injury caused by periodontitis [4, 5]. Recently, dental tissue-derived stem cell therapy has been shown to be a promising new method for the regeneration of periodontal tissue [3]. Periodontal ligament stem cells (PDLSCs), a unique population of MSCs found in periodontal ligaments, are easy to obtain and can be induced into osteoblast-like cells and adipocytes in vitro [6]. In addition, PDLSCs are currently the most favorable candidates for the treatment of advanced periodontitis, showing superiority when compared with dental pulp stem cells (DPSCs) and periapical follicular stem cells (PAFSCs) [7]. Moreover, in a swine model, researchers transplanted human PDLSCs (hPDLSCs) into alveolar bone defect areas and found that periodontal tissue was eventually repaired and regenerated [4], supporting the potential of these cells in tissue regeneration.

Stromal precursor antigen-1 (STRO-1) is a widely used marker of MSCs [8–10]. STRO-1 was found to be related to adherence, proliferation, and multilineage differentiation potential [10, 11], indicating that STRO-1(+) cells may represent a unique subgroup of hPDLSCs with specific functions in the healing of periodontitis-associated bone damage.

Parathyroid hormone (PTH), a peptide hormone produced at the endoplasmic reticulum, regulates calcium and phosphorus metabolism and mineral homeostasis [12]. A recent study showed that PTH binds to the PTH receptor on MSCs, committing MSCs to the osteoblast lineage and promoting bone formation [13]. Intermittent administration of PTH (1-34) has been shown to increase bone-to-implant contact, induce new bone formation around the implants, and increase bone mineral density [14]. Furthermore, PTH (1-34) stimulates new bone formation, leading to enhanced periodontal healing [15].

In this study, we hypothesized that PTH would affect the osteogenic capacity of PDLSCs and show the potential for application as a cell source in periodontitis treatment. Therefore, we isolated and characterized STRO-1(+) hPDLSCs, compared the levels of PTH receptor in STRO-1(+) and STRO-1(−) PDLSCs, and evaluated the effects of PTH treatment on the differentiation capacity of STRO-1(+) PDLSCs.

## 2. Materials and Methods

### 2.1. Cell Culture and PTH Treatment

Healthy premolars were collected from 10 adults (15–20 years of age; five men and five women) for orthodontic purposes at the Department of Oral and Maxillofacial Surgery, the Affiliated Stomatological Hospital of Sun Yat-sen University. All participants provided informed consent for the collection and use of their tissues. The protocols were approved by the University Ethics Committee.

PDLSCs were isolated and cultured as previously reported [6]. Briefly, periodontal tissue was gently separated from the surface of the middle third of the root and then digested with 3 mg/mL collagenase type I and 4 mg/mL dispase (Gibco-BRL, Gaithersburg, MD, USA) at 37°C for 1 h. Colony-forming cells were collected and then cultured in alpha modified Eagle medium (Gibco-BRL) supplemented with 10% fetal bovine serum (Gibco-BRL), 100 *μ*g/mL streptomycin, 100 U/mL penicillin (Hyclone, Logan, UT, USA), 200 *μ*M l-ascorbic acid (Sigma-Aldrich, St. Louis, MO, USA), and 5 mM l-glutamine (Gibco-BRL) at 37°C in an atmosphere containing 5% CO_2_. The medium was changed every 3 days. Cells were used at passages 3–5. For intermittent PTH treatment, cells were treated with 10^−12^ M PTH (1-34) (Sigma-Aldrich) for 6 h, followed by another 6 h treatment after 32 h. Cells were harvested at various times after treatment with 10^−12 ^M PTH (1-34) for RNA and protein isolation.

### 2.2. Flow Cytometry

For flow cytometry, 5 × 10^5^ cells were collected and washed with phosphate-buffered saline (PBS). Cells were then incubated with PE-labeled anti-CD90, anti-CD105, anti-CD166, and anti-CD34 antibodies (1 : 10; Becton Dickinson Biosciences, San Jose, CA, USA) and with FITC-labeled anti-CD146 antibodies (1 : 10; Life Technologies Corp., Carlsbad, CA, USA) at room temperature for 1 h in the dark. Cells were incubated with STRO-1 IgM (1 : 10; Life Technologies Corp.) at room temperature for 1 h and then incubated with PE-conjugated anti-IgM (1 : 500; Life Technologies Corp.) for 30 min in the dark. After incubation, cells were washed three times with PBS and resuspended in 300 *μ*L PBS. Flow cytometry was carried out using a BD Accuri C6 (Becton Dickinson Biosciences). Data were analyzed using CF Low Plus Software (Becton Dickinson Biosciences).

### 2.3. Pluripotency of hPDLSCs

PDLSCs (passage 3) were seeded at a density of 1 × 10^5^ cells/well in 12-well plates. For osteogenic differentiation, after reaching 70–80% confluence, cells were cultured in osteogenic-induction medium (Cyagen Biosciences Inc., Santa Clara, CA, USA) according to the manufacturer's protocol. Three weeks later, cultures were fixed with 4% paraformaldehyde for 15 min and stained with Alizarin red; quantification of mineralization was performed as previously described [16]. For adipogenic differentiation, cells were cultured in adipoinductive medium (Cyagen Biosciences Inc.) according to the manufacturer's protocol. Two weeks later, cells were fixed with 4% paraformaldehyde for 30 min and stained with Oil Red O.

### 2.4. Immunomagnetic Cell Sorting

hPDLSCs were collected at passage 3, and STRO-1(+) cells were isolated using Dynabeads rat anti-mouse IgM (Life Technologies) according to the manufacturer's instructions. Briefly, 1 × 10^7^ cells were resuspended in 1 mL PBS with 0.1% bovine serum albumin, 2 mM EDTA, and 25 *μ*L washed rat anti-mouse magnetic Dynabeads. Next, 10 *μ*L of anti-human STRO-1 IgM antibodies (Life Technologies) was added, and cells were incubated at 4°C for 1 h. The tube was then placed in a magnet for 2 min, and the bead-bound STRO-1(+) cells and bead-free STRO-1(−) cells were isolated.

### 2.5. Cell Immunofluorescence Staining

At passage 4, STRO-1(+) and STRO-1(−) hPDLSCs (1 × 10^4^) were cultivated overnight on cover slips (NEST Biotech Co. Ltd., Shanghai, China). Cells were washed with PBS, fixed in 4% paraformaldehyde at room temperature for 10 min, incubated with blocking solution containing 1% BSA for 30 min, and then incubated with primary anti-STRO-1 IgM (1 : 100; Life Technologies) and anti-PTH1R IgG antibodies (1 : 50; Cambridge, UK) overnight at 4°C. Cells were then incubated with Alexa Fluor 488-conjugated anti-mouse IgM or IgG (1 : 250; Life Technologies) for 45 min at room temperature. Cell nuclei were counterstained with DAPI (Life Technologies) and photographed with Zeiss Axio Observer Z1 (Carl Zeiss, Oberkochen, Germany).

### 2.6. Quantitative Real-Time Reverse Transcription Polymerase Chain Reaction (RT-qPCR)

Total RNA was extracted at various times using TRIzol reagent (Life Technologies), and first-strand cDNA was synthesized using a reverse transcriptase M-MLV Kit (TaKaRa, Shiga, Japan). Gene expression was quantified by RT-qPCR using a SYBR Green kit (Roche, Basel, Switzerland) with gene-specific primers for the detection of* PTH1R*, runt-related transcription factor 2 (*RUNX2*), Sp7 (also known as Osterix), and GAPDH. The cycling parameters were as follows: 95°C for 10 min, followed by 40 cycles at 95°C for 15 s, 60°C for 20 s, and 72°C for 20 s. The primers used in this study were as follows: PTH1R: forward, 5′-AGTGCGAAAAACGGCTCAAG-3′, and reverse, 5′-GATGCCTTATCTTTCCTGGGC-3′; RUNX2: forward, 5′-TGGTTACTGTCATGGCGGGTA-3′, and reverse, 5′-TCTCAGATCGTTGAACCTTGCTA-3′; SP7: forward, 5′-CCTCTGCGGGACTCAACAAC-3′, and reverse, 5′-AGCCCATTAGTGCTTGTAAAGG-3′; and GAPDH: forward, 5′-AGGTCGGAGTCAACGGATTTG-3′, and reverse, 5′-AGGCTGTTGTCATACTTCTCAT-3′. The primer pair for human* PTH1R* was designed using the Primer Premier 5.0 program based on the cDNA sequence data. The primers for* RUNX2*,* SP7*, and* GAPDH* were previously described by Sakaki-Yumoto et al. [17], Zhang et al. [18], and Nozell and Chen [19].

### 2.7. Western Blotting

Cell lysates containing 40 *μ*g total protein were loaded on sodium dodecyl sulfate polyacrylamide gels, separated by electrophoresis, and transferred to nitrocellulose membranes. Membranes were blocked with 5% nonfat dry milk for 2 h at room temperature and then incubated with anti-PTH1R (1 : 100; Boster, Wuhan, Hubei, China), anti-RUNX2 (1 : 100; Boster), anti-Sp7 (1 : 250; Sigma-Aldrich), and anti-*β*-actin (1 : 5000; Sigma-Aldrich) antibodies overnight at 4°C. Membranes were washed, incubated with IRDye 680LT goat anti-mouse or goat anti-rabbit IgG (1 : 3000; LI-COR Biosciences, Lincoln, NE, USA) for 30 min, and scanned using an Odyssey two-color infrared laser imaging system (LI-COR). Each experiment was performed in triplicate.

### 2.8. Alkaline Phosphatase (ALP) Activity

ALP activity was measured after 8 days of osteogenic differentiation. Cells were harvested with lysis buffer (20 mM Tris-HCl, 150 mM MgCl_2_, 1% Triton X-100, and 1 mM phenylmethylsulfonyl fluoride). Total protein concentrations were determined using a Pierce BCA Protein Assay Kit (Life Technologies). Fifty microliters of supernatant was added to 50 *μ*L* p*-nitrophenyl phosphate hexahydrate (1 g/L; Sigma-Aldrich) containing 1 mM MgCl_2_, and the mixture was incubated at 37°C for 30 min. Absorption at 405 nm was measured in duplicate well with a microplate reader (BioTek Instruments, Inc., Winooski, VT, USA). ALP activity per total protein (*μ*g) represented the millimoles of* p*-nitrophenol released after a 30 min incubation at 37°C.

## 3. Results

### 3.1. Characterization of hPDLSCs

**Figure 1:**
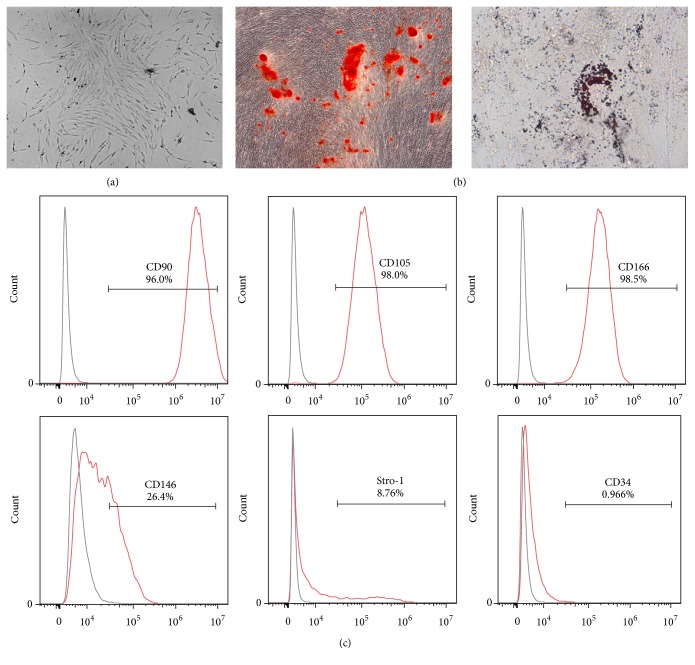
Characterization of hPDLSCs. (a) The morphology of colony-forming hPDLSCs (magnification: 50x). (b) Differentiation potential of hPDLSCs. Differentiated cells were stained with Alizarin red (left; magnification: 100x) or Oil Red O (right; magnification: 630x). (c) Immunophenotypic profiling was performed to detect CD90, CD105, CD166, CD146, Stro-1, and CD34.

hPDLSCs were isolated from the root surface of premolars and cultured. The colony-forming hPDLSCs displayed a uniform fibroblast-like morphology (Figure 1(a)). Culture-expanded hPDLSCs exhibited osteogenic and adipogenic differentiation potential, as shown by the formation of mineralized nodules and lipid-containing adipocytes (Figure 1(b)).

MSC markers on hPDLSCs were then measured by flow cytometry. The majority of the adherent cells expressed MSC markers, including CD90 (96.0%), CD105 (98.0%), CD166 (98.5%), CD146 (26.4%), and STRO-1 (8.76%), but were negative for the hematopoietic cell marker CD34 (0.966%; Figure 1(c)), suggesting that the isolated hPDLSCs were indeed stem cells of mesenchymal origin.

### 3.2. STRO-1(+) hPDLSCs Exhibited Stronger Osteogenic Ability

**Figure 2:**
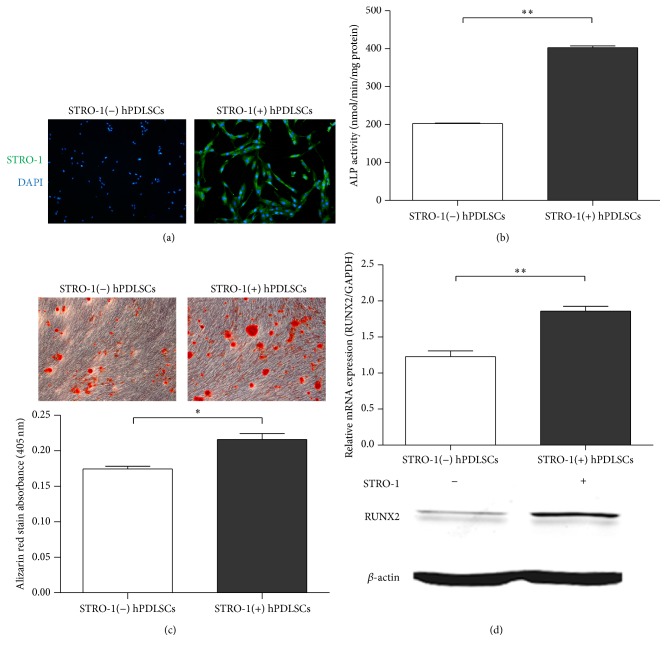
STRO-1(+) hPDLSCs exhibited strong osteogenic capacity. (a) Immunostaining for Stro-1 in isolated STRO-1(+) and STRO-1(−) hPDLSCs (magnification: 50x). The surface marker Stro-1 was stained with FITC and visualized using fluorescence microscopy. Nuclei were stained with DAPI (blue). (b) ALP activity in STRO-1(+) and STRO-1(−) hPDLSCs. (c) Osteogenic differentiated cells were stained with Alizarin red (magnification: 100x). (d) Real-time PCR and western blot analyses of Runx2 expression in STRO-1(+) and STRO-1(−) hPDLSCs. The expression of each target was normalized to that of GAPDH. Data are presented as the means ± SDs of three independent experiments performed in duplicate. ^*∗*^ *P* < 0.05, ^*∗∗*^ *P* < 0.01.

A recent study showed that STRO-1(+) cells exhibit higher adherence, proliferation, and multipotency [11]. Therefore, we next analyzed the characteristics of STRO-1(−) and STRO-1(+) hPDLSCs after isolation of both cell types. A distinct positive immunoreaction for STRO-1 was observed in STRO-1(+) hPDLSCs but not in STRO-1(−) hPDLSCs (Figure 2(a)).

We then conducted ALP activity and Alizarin red staining assays to compare the differentiation potential between STRO-1(+) and STRO-1(−) hPDLSCs. Our results showed that the ALP activity of STRO-1(+) hPDLSCs was significantly higher than that of STRO-1(−) hPDLSCs (Figure 2(b)). Alizarin red staining on day 21 revealed that the mineralized nodules of STRO-1(+) cells were larger (Figure 2(c)). These data were supported by the results of RT-qPCR analysis and western blotting (Figure 2(d)). Indeed, the expression of* RUNX2*, a typical osteogenesis-related gene, was higher in STRO-1(+) cells than in STRO-1(−) cells (Figure 2(d)). Thus, our data suggested that STRO-1(+) hPDLSCs exhibited higher differentiation potential.

### 3.3. PTH Treatment Enhanced the Osteogenesis of STRO-1(+) hPDLSCs

**Figure 3:**
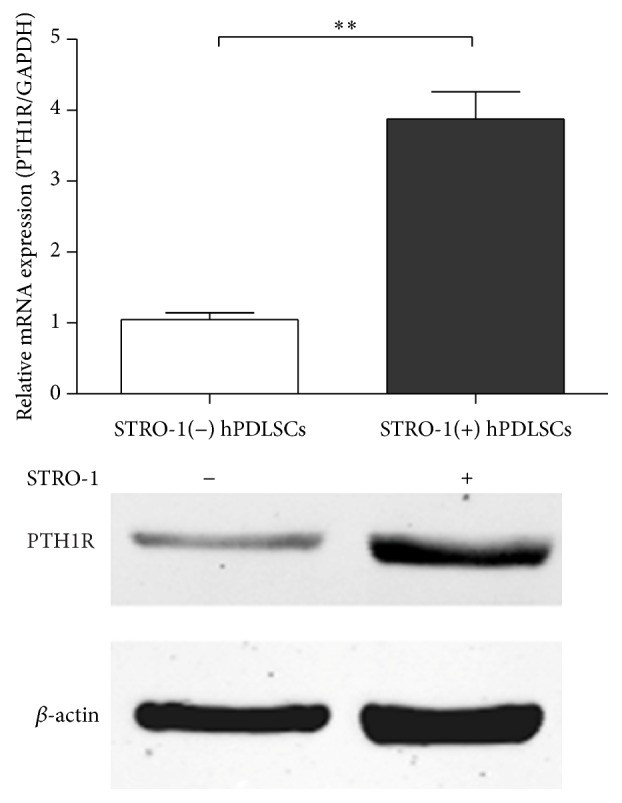
Real-time PCR and western blot analyses of PTH1R expression in STRO-1(−) and STRO-1(+) hPDLSCs. The expression of each target was normalized to that of GAPDH. Data are presented as means ± SDs of three independent experiments performed in duplicate. ^*∗∗*^ *P* < 0.01.

We next compared the expression levels of PTH1R in STRO-1(+) and STRO-1(−) hPDLSCs. Interestingly, the mRNA and protein levels of PTH1R in STRO-1(+) hPDLSCs were higher than those in STRO-1(−) hPDLSCs (Figure 3), indicating that STRO-1(+) cells may be more sensitive to PTH1R treatment, which could therefore affect the bone differentiation potential and suitability for tissue engineering.

**Figure 4:**
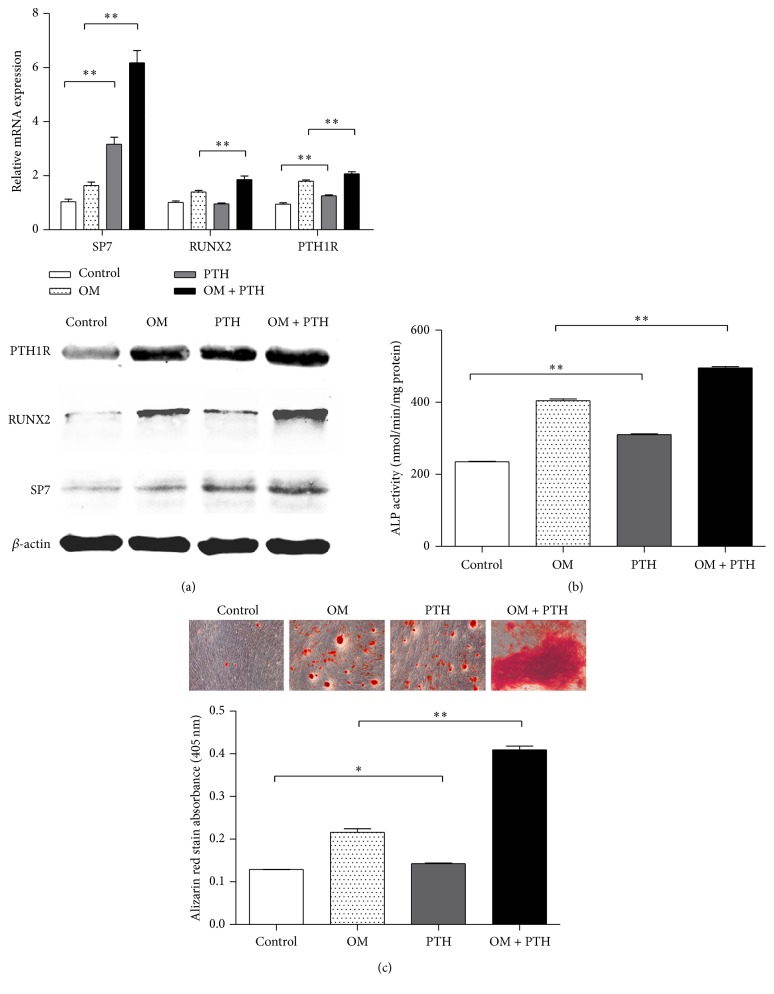
The osteogenic capacity of STRO-1(+) hPDLSCs was enhanced by PTH. (a) Real-time PCR and western blot analyses of PTH1R, RUNX2, and SP7 expression in STRO-1(+) hPDLSCs before and after PTH treatment and induction by osteogenic-induction medium (OM). The expression of each target was normalized to that of GAPDH. (b) ALP activity in STRO-1(+) hPDLSCs. (c) Cells subjected to osteogenic differentiation were stained with Alizarin red (magnification: 100x). Data are presented as the means ± SDs of three independent experiments performed in duplicate. ^*∗*^ *P* < 0.05, ^*∗∗*^ *P* < 0.01.

To test this hypothesis, we treated the cells with PTH intermittently and detected changes in the expression of osteogenesis-related genes. After 8 days of PTH treatment and osteogenic induction, the expression levels of osteogenesis-related genes were quantified by RT-qPCR and western blotting. After induction, the expressions of* PTH1R*,* RUNX2*, and* SP7* were upregulated by PTH treatment (Figure 4(a)). Moreover, intermittent PTH treatment elevated* SP7* expression in both induced and uninduced cells, and PTH increased the expression of* PTH1R*, creating a positive feedback loop (Figure 4(a)).

We then used ALP activity assays to determine whether PTH affected osteogenic differentiation. PTH-treated cells exhibited stronger osteogenic differentiation potential (*P* < 0.01; Figure 4(b)). After 22 days of induction, the formation of mineralized nodules was observed and measured. Control cells formed small mineralized nodules in terms of both area and density. In contrast, in cells treated with osteogenic medium and PTH, the formation of mineralized nodules was significantly increased in terms of both area and intensity (Figure 4(c)). Taken together, our data showed that intermittent treatment with PTH significantly enhanced osteogenesis in STRO-1(+) hPDLSCs.

## 4. Discussion

In this study, we aimed to determine whether PTH affected the osteogenic capacity of STRO-1(+) PDLSCs in order to analyze the potential applications of these cells in the treatment of periodontitis. Our results showed that PTH could stimulate osteogenesis in STRO-1(+) hPDLSCs, suggesting that this population of cells could be used in tissue regeneration to repair periodontitis-dependent bone damage.

We isolated PDLSCs from periodontal ligaments and evaluated their biological and immunological properties. Colony-forming cells expressed the surface markers CD90, CD105, and CD166 at high frequencies (95%) but rarely expressed CD34 (less than 2%). Moreover, the cells exhibited osteogenic and adipogenic differentiation potential. These results indicated that we had successfully isolated PDLSCs, according to the criteria presented in the International Society for Cellular Therapy position statement [6, 20]. With the capacity for self-renewal and differentiation into osteoblast-like cells, PDLSCs have become a promising source for regeneration of tissue in the treatment of bone defects [4, 21].

STRO-1(+) cells exhibited a more primitive phenotype [22, 23]. We found that 8.76% of total hPDLSCs were STRO-1(+), consistent with previous studies (6.7–20%) [24–26]. The purified STRO-1(+) cells exhibited higher differentiation potential, supporting the hypothesis that STRO-1(+) and STRO-1(−) cells represent different subgroups of cells with different functions in differentiation [27, 28]. Similarly, in previous studies, the osteogenic ability of STRO-1(+) hPDLSCs was found to be significantly higher than that of unsorted cells [29, 30]. In particular, we found that STRO-1(+) hPDLSCs expressed higher levels of PTH1R, suggesting that these cells may be a unique phenotype that are more sensitive to PTH treatment.

Previous studies have found that intermittent or low-dose PTH (1-34) enhances bone repair and increases bone turnover [31, 32]. In our study, we used human STRO-1(+) PDLSCs as a model to explore the roles of PTH in osteogenesis. We found that PTH (1-34) increased the expression of osteoblast-related genes and the mineralization capacity of the cells, supporting the future applications of PTH in periodontitis treatment. Further studies are needed to examine the effects of PTH on periodontal tissue regeneration in vivo by transplantation of PTH-treated STRO-1(+) PDLSCs into alveolar bone defect areas in an appropriate animal model.

The functions of PTH are mediated by a G protein coupled receptor, referred to as PTH1R, the PTH-1 receptor, which regulates skeletal development, bone turnover, and mineral ion homeostasis [33, 34]. In continuous treatment, PTH caused severe bone loss and inhibits bone related protein expression [35, 36], while intermittent administration of PTH enhanced bone formation [37]. These studies have shown that PTH may be a double-edged sword, suggesting that the molecular mechanisms of PTH may be highly complicated [38]. The time-dependent action of PTH might be due to differences in the signal transduction systems, indicating a synchronized regulation of cAMP/PKA and PKC signaling after PTH stimulation [39]. While the PKA pathway acts in response to short exposure to PTH, the PKC pathway is mainly involved at longer times of exposure in an antagonist mode [40, 41]. In our study, STRO-1(+) PDLSCs were treated under the intermittent administration of PTH, which might be mediated predominantly by cAMP/PKA pathway. Interestingly, our data showed that PTH upregulated its receptor PTH1R in STRO-1(+) PDLSCs, which may result in the establishment of a positive feedback loop. The observed effects could be explained by the feedback of PKA signals on PTH1R expression levels, ligand sensitivity, and crosstalk with other downstream signaling pathways [42]. However, the nature of interactions between PTH responsive signaling systems in STRO-1(+) PDLSCs remains to be further elucidated.

Runx2 is a key transcription factor involved in the process of osteogenesis and directs multipotent mesenchymal cells to the osteoblast lineage [43]. During the process of osteogenesis, Sp7 has proved to be a direct downstream target of RUNX2, and these two proteins physically interacted and synergistically activated osteogenic genes [44, 45]. In our current study, PTH (1-34) treatment leads to a significant increase of RUNX2 and Sp7 after osteogenic induction. The anabolic effects of PTH on osteoblast-specific transcription factors (Runx2 and Sp7) could be attributed to the predominant activation of cAMP/PKA signal transduction [46–48]. However, our data showed that the enhancement of Sp7 expression by PTH was even more robust in both uninduced and induced STRO-1(+) cells than that of Runx2, indicating a Runx2 independent activation of Sp7 gene expression [49]. It is possible that other genes upstream of Sp7 may also be affected by PTH, or effects of PTH may be directly mediated through Osx and independently of Runx2 [50, 51]. Further studies are needed to fully elucidate the specific mechanisms involved in these processes.

In summary, we achieved successful culture of hPDLSCs and separation of STRO-1(+) and STRO-1(−) cells. Our additional experiments suggested that STRO-1(+) hPDLSCs had higher sensitivity to PTH and stronger osteogenic ability, which may play a major role in bone regulation in the presence of PTH. These data provide a deeper understanding of the effects of PTH on the regeneration of periodontal support tissues and may facilitate the development of improved biomaterials for periodontal stem cell treatment.

## 5. Conclusion

STRO-1(+) hPDLSCs represented a more promising cell resource for biomaterials and tissue engineering applications. Intermittent PTH treatment improved the capacity of STRO-1(+) hPDLSCs to repair damaged tissue and ameliorate the symptoms of periodontitis. Our results provided evidences for applications of PTH in stem cell therapy for periodontitis diseases.
